# Antral membrane balloon technique versus Densah bur in crestal sinus lift with simultaneous implant placement: a randomized clinical trial

**DOI:** 10.1186/s12903-024-04609-8

**Published:** 2024-08-08

**Authors:** Mohamed Mahmoud Alajami, Heba Abo-Elfetouh Elsheikh, Shaimaa Ahmed Abo El-Farag, Noha Ahmed Mansour

**Affiliations:** 1 Department of Oral and Maxillofacial Surgery, Faculty of Dentistry, Mansoura University, Mansoura, Egypt; 2 Department of Fixed Prosthodontics, Faculty of Dentistry, Mansoura University, Mansoura, Egypt

**Keywords:** Allograft, Antral membrane balloon elevation, Crestal sinus lift, Densah Bur, Posterior maxilla, Simultaneous Implant Placement

## Abstract

**Background:**

The posterior maxilla presents challenges for implant insertion because of the poor bone quality as well as the loss of vertical bone height. Indirect transcrestal sinus lift techniques are advised when a few millimeters of additional height are needed. This study aimed to evaluate the clinical and radiographic outcomes of antral membrane balloon technique versus Densah burs for transcrestal maxillary sinus lifting with simultaneous implant placement.

**Materials and methods:**

This randomized clinical trial was conducted on 22 patients received 32 dental implants for replacement of missed maxillary posterior teeth after crestal maxillary sinus lifting. The patients were randomly divided into two groups. Group 1, patients underwent crestal sinus floor elevation with simultaneous implant placement using antral membrane balloon technique. Group 2, patients underwent crestal sinus floor elevation with simultaneous implant placement using Densah burs. Patients were evaluated clinically and radiographically using cone beam computed tomography (CBCT) at regular time intervals immediately, 6 months and 12 months after surgery. All clinical and radiographic parameters were statistically analyzed.

**Results:**

All dental implants were successful for 12 months of follow up. Regarding implant primary stability, there was a statistical significant difference between the study groups in favor of Densah group (*P* = 0.004), while there was no significant difference after 6 months (*P* = 0.07). Radiographically, balloon group showed a statistically significant immediate postoperative vertical bone height (*P* < 0.0001), and significant reduction in vertical bone height after 6 months (*P* < 0.0001). Densah group showed significant increase in bone density (*P* ≤ 0.05).

**Conclusion:**

Both techniques demonstrated successful clinical and radiographic outcomes for crestal sinus lift. The antral membrane balloon group demonstrated better immediate postoperative vertical bone gain, while Densah burs had higher implant primary stability and bone density.

**Trial registration:**

This study was registered in Clinical-Trials.gov PRS (https://register.clinicaltrials.gov) under identification number NCT05922592 on 28/06/2023.

## Background

Dental implants have become the preferred method for replacing missing teeth. However, the process of placing dental implants in posterior maxilla presents a significant challenge caused by atrophy of maxilla after tooth extraction and the pneumatization of the maxillary sinus, reducing the amount of bone available for implant placement [1]. These factors, combined with the generally inferior quality of bone in this area, can complicate the process of insertion of dental implant. Therefore, it is necessary to use a longer implant to counteract these issues and enhance the stability of the dental implant [2, 3].

Various treatment options have been suggested for a vertically deficient edentulous posterior maxilla including direct sinus elevation through a lateral window access and indirect sinus elevation through a crestal approach [4]. The lateral window method delivers a reliable clinical result. Nevertheless, its drawbacks, such as invasiveness, morbidity, the risk of damaging the alveolar antral artery, sinus membrane perforation, delayed healing, and a higher chance of postoperative infection, are significant. In contrast, the less invasive transcrestal indirect sinus elevation procedures require less time and have a lower morbidity rate [5].

Several surgical techniques for indirect elevation of the sinus membrane have been developed. In 1994, Summers [6] introduced the osteotome sinus floor elevation (OSFE) technique, which involves the use of an osteotome to fracture the sinus floor and lift the sinus membrane, facilitating the placement of graft materials and implants in the subantral space through the osteotomy site. However, this procedure has drawbacks, including exposure to the explosive force of the maxilla with inadequate control, inadvertent displacement or fracture, membrane perforation, benign paroxysmal positional vertigo, and patient pain [7].

The antral membrane balloon elevation (AMBE) procedure has been increasingly utilized as a minimally invasive technique for sinus lifting procedures [8]. This method utilizes an inflatable balloon to evenly elevate the sinus membrane with minimal trauma and is particularly effective in hard-to-reach areas. It involves a limited incision, minimal mucoperiosteal flap reflection, and less risk of sinus membrane perforation [9–13].

In 2017, Huwais and Meyer [14] presented a novel osseodensification approach; using Densah burs instead of conventional drills to prepare the implant site. It has several benefits, one of which is the increased implant-bone contact achieved through compaction autografting, this is mostly due to the viscoelastic properties of bone, which cause time-dependent stress to result in time-dependent strain.

Huwais et al. [15] introduced the use of Densah burs for maxillary sinus lifting, using the benefits of the osseodensification concept. This is based on the idea that a unique flute design in a densifying, non-cutting mood with counterclockwise motion and irrigation causes a hydraulic wave at the bur’s apex. This wave pushes the sinus membrane upward, and it also causes the Schneiderian membrane to be elevated with a reduced risk of perforation when grafting material is present. Consequently, this method is recommended to offer a safe, less invasive way to elevate the maxillary sinus with fewer complications than the osteotome or lateral approach [15].

The purpose of this study was to compare the clinical and radiographic outcomes of antral membrane balloon elevation versus Densah burs as minimally invasive approaches for transcrestal maxillary sinus lifting with simultaneous implant placement. The primary objectives were to assess vertical bone gain and reduction in vertical bone height, and the secondary objectives were to assess postoperative pain, implant stability and relative bone density.

The null hypothesis of this study is that: there is no significant difference between using the antral membrane balloon elevation and the Densah burs for transcrestal maxillary sinus lifting with simultaneous implant placement regarding pain, implant stability, vertical bone gain, reduction in vertical bone height and bone density.

## Materials and methods

### Patient selection

22 patients seeking replacement of missed maxillary posterior teeth via dental implants and having pneumatized maxillary sinus at the planned implant sites were selected to perform a crestal sinus lift procedure with simultaneous implant placement. All patients were selected from the outpatient clinic of the Oral and Maxillofacial Surgery Department, Faculty of Dentistry, Mansoura University.

Every patient was informed about possible expected risks and benefits of the planned surgery, and a written informed consent was obtained from all included individuals before the surgical procedure. The Institutional Review Board (IRB) of the Faculty of Dentistry, Mansoura University, Mansoura, Egypt, approved the current study in compliance with the seventh revision of the Helsinki Declaration in 2013 under protocol number A08030123 and registered in Clinical-Trials.gov PRS (https://register.clinicaltrials.gov) under identification number NCT05922592 on 28/06/2023.

### Criteria for patient selection

**Table:** 

Inclusion criteria	Exclusion Criteria
1. Patients with one or more missed posterior maxillary teeth and a residual bone height (RBH) of 4–7 mm between the alveolar ridge crest and the floor of the maxillary sinus at the planned implant site.	1. Patients with systemic disease that contraindicate the surgical procedure as uncontrolled diabetes mellitus, bleeding disorders, recent myocardial infarction, head and neck radiation, and mental disorders
2. Adequate width of alveolar ridge to accommodate dental implant (6 mm or more).	2. Patients taking systemic drugs that could affect bone healing as immune suppressive drugs and bisphosphonates.
3. Patients age range from 20 to 50 years old from both sexes.	3. Any pathological condition at the site of surgery.
4. Adequate inter arch space of at least 8 mm.	4. Patient with repeated maxillary sinusitis or previous sinus surgery.
5. Good oral hygiene.	5. Smoking and alcoholism.
6. Patent ostium of the maxillary sinus.	6. Parafunctional habits such as bruxism and clenching.

### Sample size calculation

The sample size was calculated based on mean vertical bone gain retrieved from a previous study by Hashem et al. [16] Using G power program version 3.1.9.7 to calculate sample size based on effect size of 1.25 using 2-tailed test, α error = 0.05 and power = 90.0%, the total calculated sample size was 15 implants in each group at least.

### Study design

This prospective randomized clinical trial adhered to CONSORT guidelines for conducting clinical trials (CONSORT Flowchart). The patients in this trial were randomly split up into two groups:

#### Group 1

16 implants were inserted in 10 patients by using antral membrane balloon elevation technique for crestal sinus floor elevation.

#### Group 2

16 implants were inserted in 12 patients by using Densah burs for crestal sinus floor elevation.

**Figure:**
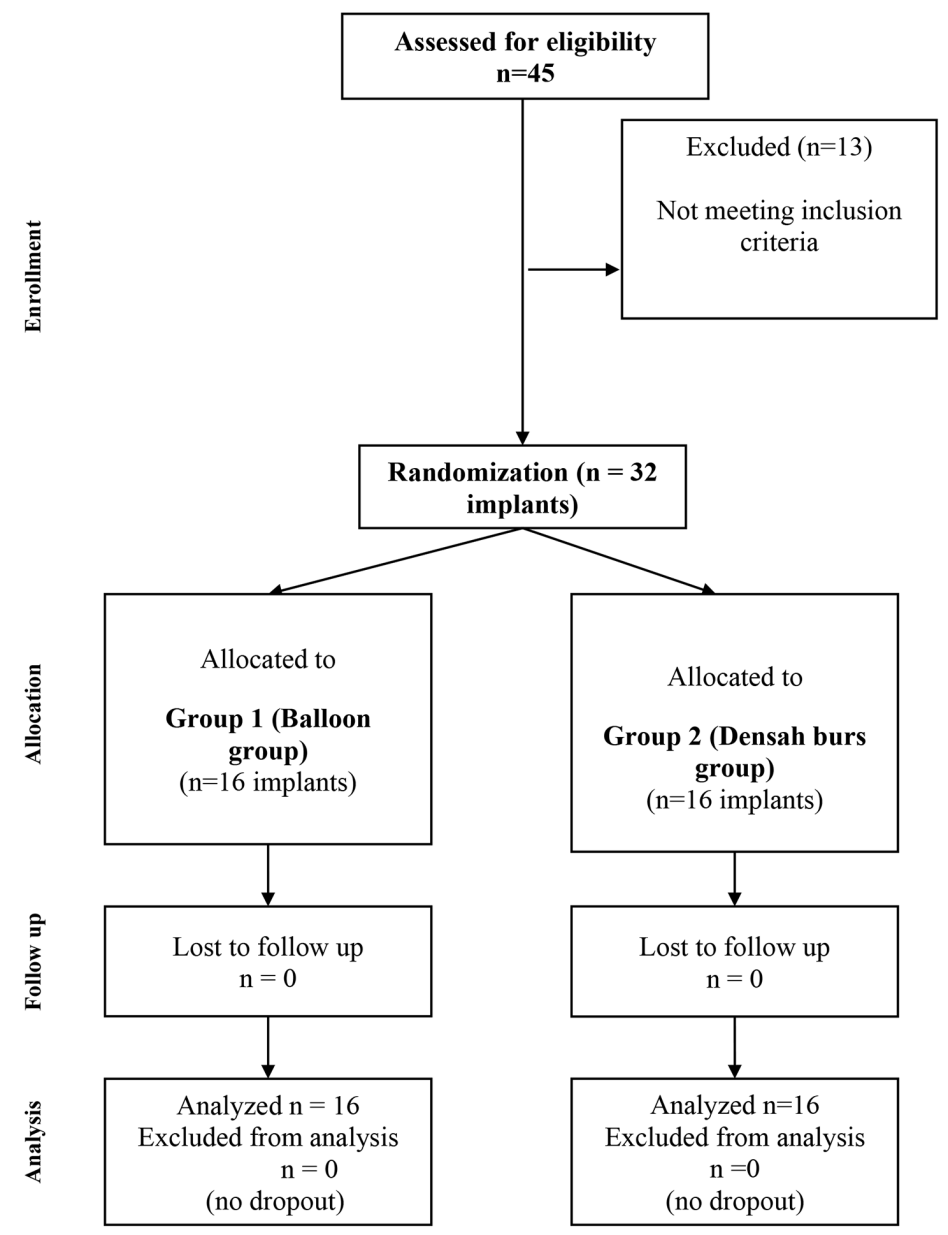
Flowchart diagram of the study protocol according to Consort guidelines

### Randomization

The randomization was done by using a computer-generated randomization list (SPSS v25.0). The involved edentulous spaces requiring dental implant replacement with associated sinus lift were randomly divided into two equal groups, with 16 implants for each group. Group 1 had undergone crestal sinus lift via antral membrane balloon elevation technique, while Group 2 had undergone crestal sinus lift via Densah burs.

### Preoperative measures

**Fig. 1:**
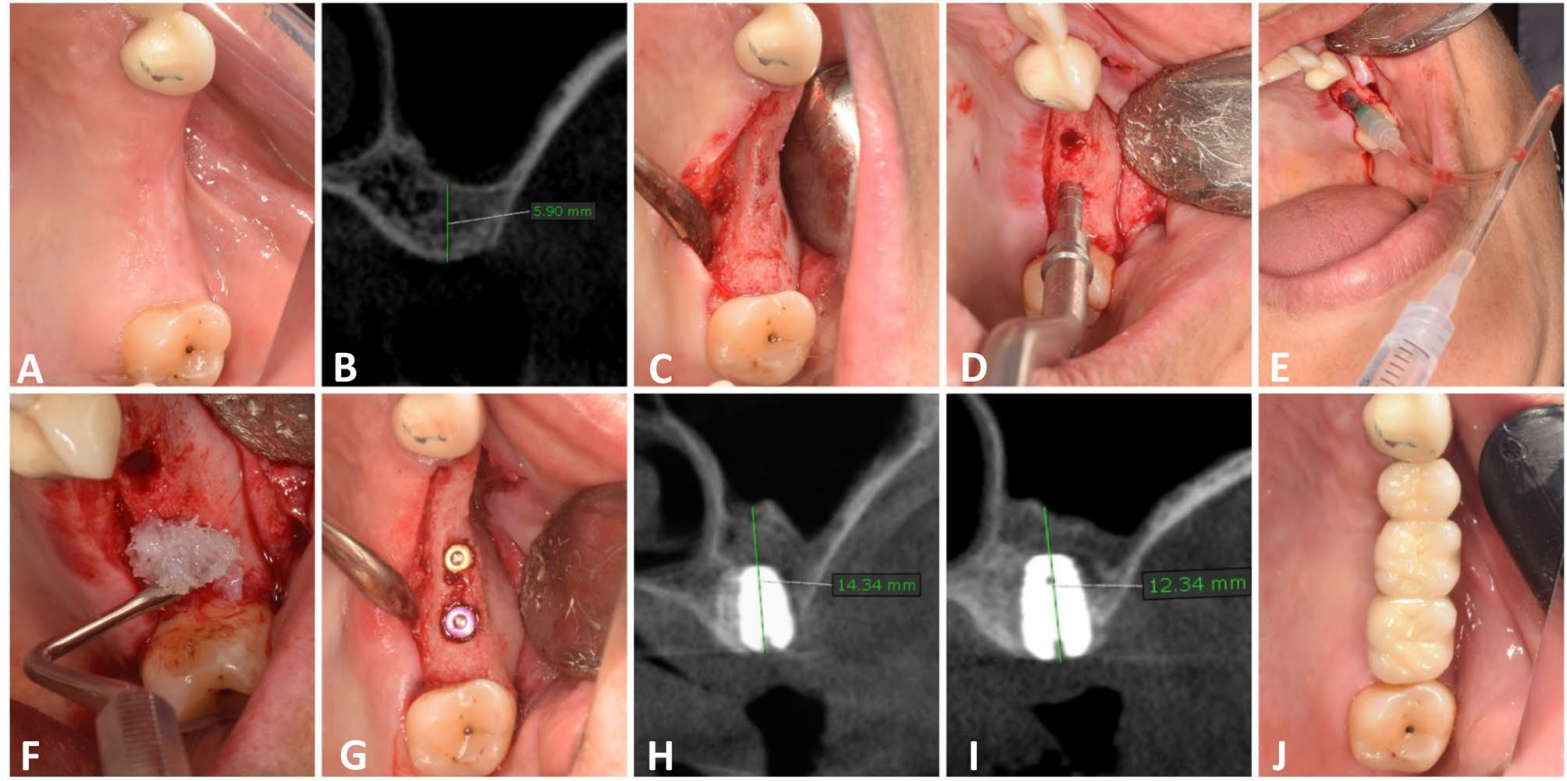
Balloon Group. **A**: A photograph showing preoperative clinical view, **B**: Preoperative CBCT showing residual bone height, **C**: Full thickness mucoperiosteal flap, **D**: Osteotome application, **E**: Insertion of the sinus balloon for lifting of the sinus membrane, **F**: Allograft application, **G**: Implants after placement, **H**: Immediate postoperative CBCT showing vertical bone height, **I**: CBCT after 6 months, **J**: occlusal view of final zirconia crown

**Fig. 2:**
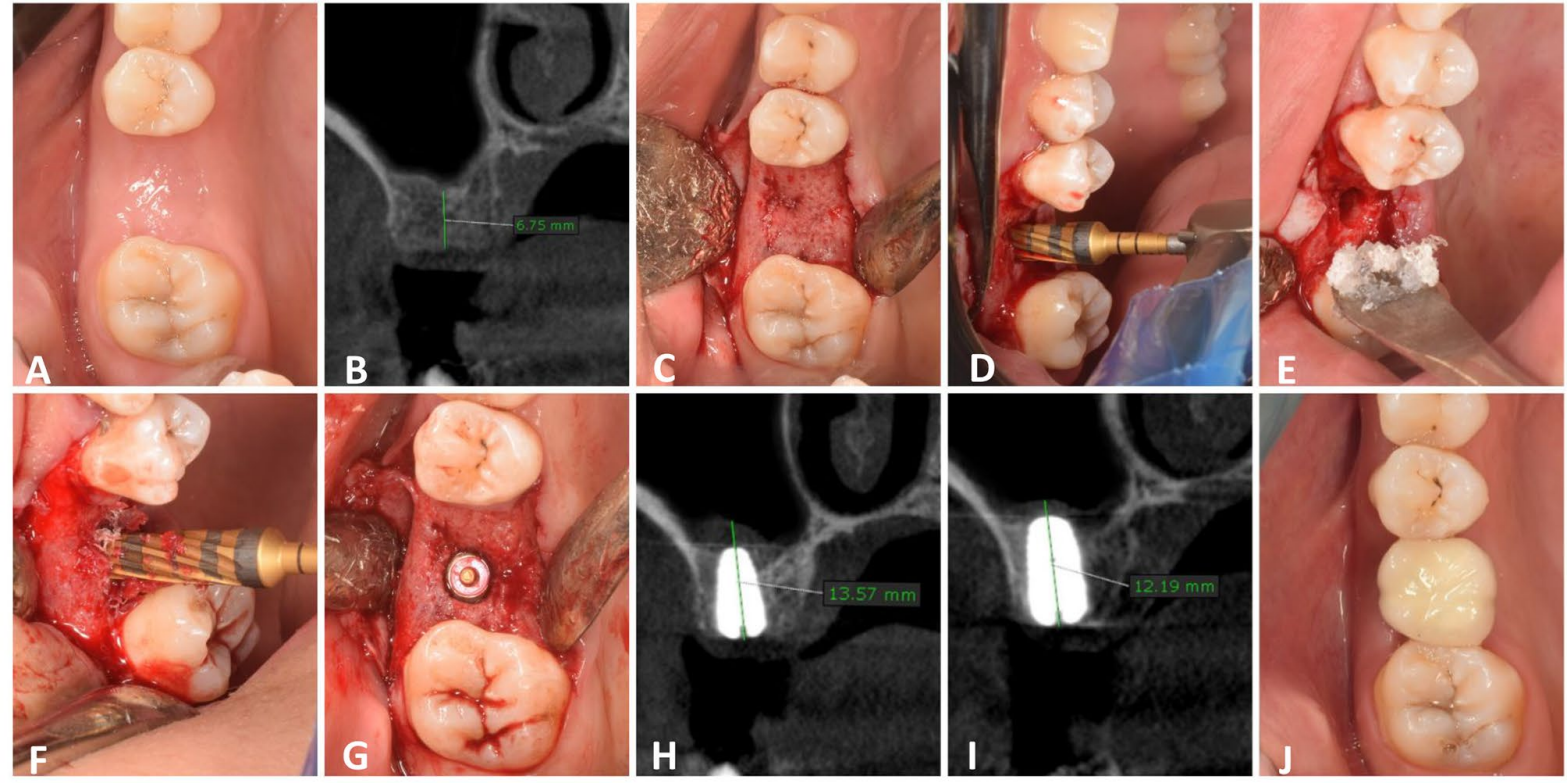
Densah Bur Group. **A**: Preoperative clinical view, **B**: preoperative CBCT, **C**: Full thickness mucoperiosteal flap, **D**: Densah sinus floor elevation, **E** & **F**: Allograft application, **G**: Implants after placement, **H**: Immediate postoperative CBCT showing vertical bone height, **I**: CBCT after 6 months, **J**: occlusal view of final zirconia crown

Preoperative panoramic radiograph was performed first as a screening method. Preoperative Cone beam computed tomography (CBCT) (Planmeca, ProMax^®^ 3D Max, Helsinki, Finland) was performed when the patient was considered as a candidate for crestal sinus lift to evaluate the RBH, the bucco-lingual and mesio-distal width of edentulous space and to detect any abnormal maxillary sinus conditions (anatomical or pathological) (Figs. 1B and 2B).

All patients were instructed to take Amoxicillin and Clavulanic acid 1gm tablets (Hibiotic, Amoun Pharmaceutical, S.A.E.) every six hours as a prophylactic antibiotic measure, 48 h prior to the surgical procedure. In addition, patients were instructed to rinse with Chlorohexidine HCl (0.12%) (Hexitol, the Arab Drug Company, Cairo, A.R.E.) twice a day.

Study Casts and Preoperative intraoral photographs were taken for each patient as a baseline record (Figs. 1A and 2A).

### Surgical procedures

Following buccal and palatal infiltration of local anesthesia (articaine 4% with epinephrine: 100.000), (ARTINIBSA, Inibsa, Spain.), a paracrestal incision was made and a full thickness mucoperiosteal flap was reflected to expose the ridge (Figs. 1C and 2C).

The initial osteotomy was performed at the planned implant site with a pilot drill up to 1 mm below the sinus floor. A guide pin was then placed in the osteotomy site to confirm the position and angulation of the osteotomy, which was verified using a digital periapical radiograph (Digora, Sordex, Finland).

### For Group 1 (Antral Membrane Balloon Elevation Group)

The implant site was drilled using a low-speed, high-torque contra-angle handpiece set to a speed of 800 to 1200 rpm. The osteotomy site was sequentially widened to the appropriate width. The remaining 1 mm of subantral bone was fractured using a graduated osteotome (Medesy Srl, Italy) and gentle tapping with a surgical mallet (Fig. 1D).

A latex balloon (Dentium sinus balloon, Korea) was attached to a catheter to be inflated and tested before being placed in the subantral space. After insertion, the balloon was filled with 1 ml of sterile saline solution and progressively inflated in a slow and controlled manner. The balloon was left inflated for 5 min to reduce the sinuses’ ability to recoil before being removed (Fig. 1E). The sinus membrane is raised by 6 mm in each 0.5 cc of saline according to the manufacturer’s instructions, so the sinus membrane was detached to the desired height in each case. The integrity of the sinus membrane was evaluated by gently performing a Valsalva maneuver and asking the patient to blow through the nose while looking for mist on a mirror.

Finally, 0.5 cc of allograft (lyo 105, lyoplast-S"^®^ for dentistry, Russia) was wetted in sterile saline solution on a sterilized dish and condensed through the osteotomy site under elevated sinus membrane using an osteotome (Fig. 1F).

### For Group 2 (Densah Burs Group)

Once the position of the guide pin was verified, Densah burs (Versah LLC, USA) were used in the recommended sequence following the protocol specified in the densification guide recommended by the manufacturer for the implant system to be utilized. The smallest Densah bur was used in a counterclockwise motion with a drill speed of 800 rpm. When resistance was met due to increased bone density, the speed was increased gradually to no more than 1500 rpm. The bur was advanced in the osteotomy up to 0.5–1 mm below the sinus floor following the path created by the pilot drill. The next wider Densah bur was used to breach the sinus floor. When feeling the haptic feedback of the bur reaching the dense sinus floor, pressure was modulated with a gentle pumping motion to advance past the sinus floor in 1 mm increments, with care not to advance it more than 3 mm beyond the sinus floor to avoid tearing the sinus membrane. With this approach, autogenous bone chips from the osteotomy get packed around at the tip of the bur, facilitating the gentle separation and lifting of the membrane (Fig. 2D).

The sequential wider Densah burs were used in Densifying Mode at 800–1500 rpm with copious irrigation and pumping motion to achieve additional width with maximum membrane lift of 3 mm (in 1 mm increments) to reach final desired width for implant placement.

After reaching the final planned osteotomy diameter, the osteotomy site was filled with 0.5 cc allograft (Fig. 2E). The final Densah bur was used in densifying mode with a low speed of 150–200 rpm and no irrigation to push the allograft toward the sinus. Employ the Densah bur to aid in compacting the allograft to lift the sinus membrane and not advance more than 2–3 mm past the sinus floor (Fig. 2F).

### For both groups

Implants fixture (NucleOSS Dental implant, T6 implant, Turkey) of proper length was guided into its proper place using light, steady finger pressure. The implants were installed 1 mm below the alveolar crestal bone using a coupling wrench with ratchet.

Resonance frequency analysis (RFA) was used to measure implant stability by utilizing an Osstell Mentor device (Osstell, Integration Diagnostics, Savadaled, Sweden). The smart peg was attached to the dental implant. The outcomes were presented as the implant stability quotient (ISQ).

Cover screws were then attached to implant fixtures (Figs. 1G and 2G). After repositioning the flap, primary closure was achieved using a combination of horizontal mattress and simple interrupted 4/0 polyproplene suture (Ghatwary Medical GMS., Egypt). An immediate postoperative CBCT was performed (within 1 week).

### Postoperative care and instructions

Patients were instructed to apply cold packs extra-orally for the first 24 h of the operation, maintain good oral hygiene measures and avoid sneezing, nose blowing or other actions that might create high intranasal pressure or vacuum.

Patients were instructed to continue with the prescribed antibiotic 2 times daily for seven days after surgery. Ibuprofen 400 mg as analgesic, for pain relief if needed (Brufen 400 mg, Abbott India Ltd). Chymotrypsin as anti-inflammatory (Alphintern, Amoun Pharmaceutical Co Egypt S.A.E) 0.375 mg 3 times daily for 7 days. Patients were also instructed to rinse with Chlorohexidine HCl (0.12%) (Hexitol, the Arab Drug Company, Cairo, A.R.E.) for 2 weeks. Xylometazoline as nasal decongestant (Otrivin 1%, Novartis, Germany) 2 drops into each nostril up to 3 times a day for 3–5 days. Following this course of treatment, the sutures were removed after one week.

### Second stage surgery

Six months later, a second stage surgery was carried out. The surgical cover screw was exposed and replaced by a healing abutment for 14 days.

### Prosthetic rehabilitation

Using a laboratory analogue and an impression post, an indirect impression was made in order to produce a working cast. Then, the healing abutment was replaced by the functional abutment. A zirconium crown was cemented after checking occlusion and marginal fit (Figs. 1J and 2J).

## Evaluation

Each patient was regularly evaluated clinically and radiographically:

### Clinical evaluation

#### Postoperative pain

The pain was assessed at 2nd, 3rd and 7th day postoperative using a 10-point visual analogue scale (VAS; 0–1 = None, 2–4 = Mild, 5–7 = Moderate, 8–10 = Severe) [17].

#### Implant Stability

During the time of implant placement and after 6 months, the stability of the implants was evaluated by RFA with the Osstell Mentor device. The results were expressed using ISQ.

### Radiographic evaluation

CBCT was utilized for radiographic assessment preoperative, immediately postoperative and after 6 months (Figs. 1H and I and 2H and I). For standardization, all radiographic evaluation was done by a single well-trained examiner, and CBCT scans were conducted at the same radiology center (Planmeca, ProMax^®^ 3D Max, Helsinki, Finland) with identical parameters (89 kVp, 24 s, 10 mA, and a field of view of 6 cm × 8 cm), a standardized head position, maximum teeth intercuspation and a horizontal plane parallel to the floor. OnDemand3D software was employed for image processing and reconstruction. In order to provide reference points for pre- and post-surgery comparisons, fixed anatomical landmarks related to the planned implant site and the dental implant itself, were included in the two CBCT cross-sectional views. The angles and anatomical details of the two views were then matched by correcting the same sites in the two CBCT scans [18]. CBCT was used to assess the following:

1. **Residual bone height (RBH)**: was measured as the distance from the alveolar crest to the floor of the maxillary sinus at the planned implant site on preoperative CBCT.
2. **Vertical bone height (VBH)**: was measured as the distance from the alveolar crest to the new sinus floor at the middle of the implant immediately postoperative and after 6 months.
3. **Vertical bone gain (VBG)**: was evaluated by subtracting residual bone height from vertical bone height immediately postoperative and after 6 months (VBH-RBH).
4. **Implant protrusion length (IPL)**: was measured as the distance from the initial sinus floor to the implant apex immediately postoperative.
5. **Reduction in Vertical Bone Height**: was measured by subtracting vertical bone height after 6 months from immediate postoperative vertical bone height.
6. **Relative alveolar bone density**: was measured by drawing standard rectangular shape buccal and palatal at the planned implant site in the preoperative CBCT. Then, this rectangular shape was drawn along the buccal and palatal walls from the implant shoulder to the apex immediately postoperative and after 6 months. All density records were collected from the cross-sectional view of CBCT using Hounsfield units (HU) [16].

### Statistical analysis

Data management and statistical analysis were performed using the Statistical Package for Social Science (SPSS) program version 25. Data were described using minimum, maximum, mean, standard deviation, and median. Independent sample t-test was used for inter-group comparison. Paired-samples t-test was used for intra-group comparison. Repeated measures analysis of variance was used. Pair-wise comparison was done with Bonferroni correction. Statistical significance was tested at P value ≤ 0.05.

## Results

A total of 22 patients (13 female and 9 male patients), ranged in age from 28 years to 49 years with a mean of 38.5 years underwent successful crestal sins lift, receiving 32 dental implants for replacement of lost posterior maxillary teeth (premolar and molars).

The implant lengths used in this study were 10 mm (87.5%) and 8 mm (12.5%). Moreover, the implant diameters used in this study were 4.1 mm (87.5%) and 4.8 mm (12.5%). All patients received zirconium crown restorations.

Fortunately, throughout 12 months of postoperative follow up period, all implants osseointegrated successfully and were clinically stable. None of the implants were lost during the whole follow up period. No complications were recorded clinically or radiographically such as infection or Schneiderian membrane perforation.

### Clinical evaluation

#### Pain score

There was no statistically significant difference in the pain score between the two groups on the postoperative days of assessment. (*P* = 0.13, 0.151 and 0.00 for 2nd, 3rd and 7th day postoperative respectively).

In each group, repeated measures analysis showed a statistically significant decrease in the pain score between the different points of measurement (*P* < 0.001) (Table 1).

**Table 1:** Pain score

Pain Score	Group		Test of significance *P*-value
	Densah Group (*n* = 16)	Balloon Group (*n* = 16)	
**Second day**			
- Min. – Max. - Median	4.00–5.00 4.00	4.00–6.00 5.00	*P* = 0.13 NS
**Third Day**			
- Min. – Max. - Median	3.00–4.00 3.00	3.00–4.00 4.00	*P* = 0.151 NS
**Seventh day**			
- Min. – Max. - Median	0.00–0.00 0.00	0.00–0.00 0.00	NA
**Test of intra-group significance** ***P*** **-value**	*P* < 0.001*	*P* < 0.001*	

Min-Max: Minimum – Maximum

*Statistically significant (*P* ≤ 0.05)

NS: Statistically not significant (*P* > 0.05)

NA: Not applicable

#### Implant stability

There was a statistically significant higher primary stability in Densah group compared with balloon group immediately postoperatively (*P* = 0.004). After 6 months, there was no statistically significant difference in the implant stability between the two groups (*P* = 0.07) (Table 2).

**Table 2:** Implant Stability

Stability	Group		Test of significance *P*-value
	Densah Group (*n* = 16)	Balloon Group (*n* = 16)	
**Postoperative**			
- Mean ± SD	68.63 ± 2.26	65.00 ± 4.21	t = 3.03 *P* = 0.004*
**At loading**			
- Mean ± SD	73.88 ± 3.79	71.50 ± 3.59	t = 1.82 *P* = 0.07 NS
**Paired t-test of significance**			
***P*** **-value**	*P <* 0.001*	*P <* 0.001*	

n: Number of implants

SD.: Standard Deviation

t-test = independent t test

*Statistically significant (*P* ≤ 0.05)

NS: Statistically not significant (*P* > 0.05)

### Radiographic evaluation

#### Residual bone height (RBH)

The mean of RBH before surgery was 6.09 ± 0.82 for group 1 and 5.95 ± 0.78 for group 2 with no statistically significant difference between the two groups (*P* = 0.624) (Table 3).

**Table 3:** The Vertical Bone Height (mm)

Vertical Bone Height (mm)	Group		Test of significance *P*-value
	Densah Group (*n* = 16)	Balloon Group (*n* = 16)	
**RBH**			
- Min. – Max.	5.20-7.00	5.00–7.00	t = 0.495
- Mean ± SD	5.95 ± 0.78	6.09 ± 0.82	*P* = 0.624 NS
**Immediate Postoperative**			
Min. – Max. - Mean ± SD	10.40–14.70 12.70 ± 1.28	13.50–15.20 14.40 ± 0.53	t = 4.9 *P* < 0.0001*
**After six months**			
- Min. – Max. - Mean ± SD	10-13.70 11.58 ± 1.08	10.1–12.90 12.11 ± 0.71	t = 1.64 *P* = 0.11 NS
**Repeated Measure ANOVA**			
***P*** **-value**	*P* = 0.001*	*P* = 0.001*	

n: Number of implants

Min-Max: Minimum – Maximum

t-test = independent t test

*Statistically significant (*P* ≤ 0.05)

NS: Statistically not significant (*P* > 0.05)

#### Vertical Bone Height (VBH)

Regarding immediate postoperative bone height, there was a statistically significant difference between the two groups in favor of balloon group (*P* < 0.0001), while after 6 months, there was no statistically significant difference between the two groups (*P* = 0.11) (Table 3).

#### Vertical Bone Gain (VBG)

There was a statistically significant higher vertical bone gain in balloon group compared with Densah group (*P* < 0.0001) immediately postoperative, while after 6 months, there was no statistically significant difference in vertical bone gain between the two groups (*P* = 0.25) (Table 4).

**Table 4:** The Vertical Bone Gain (mm)

Vertical bone gain VBG (mm)	Group		Test of significance *P*-value
	Densah Group (*n* = 16)	Balloon Group (*n* = 16)	
**Immediate Postoperative**			
- Min. – Max. - Mean ± SD	5.20–8.70 6.75 ± 1.13	7.70–8.90 8.31 ± 0.44	t = 5.15 *P* < 0.0001*
**After six months**			
- Min. – Max. - Mean ± SD	4.50–7.70 5.63 ± 1.03	4.50–7.20 6.02 ± 0.85	t = 1.168 *P* = 0.25 NS
**Paired t-test of significance**			
***P*** **-value**	*P <* 0.001*	*P* < 0.001*	

Min-Max: Minimum – Maximum

SD.: Standard Deviation

t-test = independent t test

*Statistically significant (*P* ≤ 0.05)

NS: Statistically not significant (*P* > 0.05)

#### Implant Protrusion length (IPL)

There was no statistically significant difference in the IPL between the two groups (*P* = 0.689) (Table 5).

**Table 5:** Implant protrusion length (IPL) (mm)

Implant protrusion in the sinus (IPL)	Group		Test of significance *P*-value
	Densah Group **(n = 16)**	Balloon Group **(n = 16)**	
- Mean ± SD	3.80 ± 1.07	3.66 ± 0.88	t = 0.404 *P* = 0.689 NS

n: Number of implants

SD.: Standard Deviation

t-test = independent t test

NS: Statistically not significant (*P* > 0.05)

#### Reduction in Vertical Bone Height

The reduction in vertical bone height after 6 months was statistically significant higher in the balloon group compared with the Densah group (*P* < 0.0001) (Table 6).

**Table 6:** Reduction in Vertical Bone Height (mm) after 6 months postsurgically

Reduction in Vertical Bone Height (mm)	Group		Test of significance *P*-value
	Densah Group **(n = 16)**	Balloon Group **(n = 16)**	
- Min. – Max. - Mean ± SD	0.20–1.50 1.13 ± 0.40	1.10–3.20 2.28 ± 0.57	t = 6.6 *P* < 0.0001*

Min-Max: Minimum – Maximum

SD.: Standard Deviation

t-test = independent t test

*: Statistically significant (*P* ≤ 0.05)

#### Relative bone density

Regarding buccal bone density, there was no statistically significant difference between the two groups preoperatively (*P* = 0.256), but there was a statistically significant higher buccal bone density around the implant in Densah group compared with balloon group immediately postoperative and after 6 months (*P* < 0.0001) (Table 7).

**Table 7:** Buccal and palatal bone density (HU)

Buccal Bone density	Group		Test of significance *P*-value
	Densah Group (*n* = 16)	Balloon Group (*n* = 16)	
**Preoperative** - Mean ± SD	290.75 ± 90.75	260.50 ± 52.01	t = 1.16 *P* = 0.256 NS
**Immediate postoperative** - Mean ± SD	740.63 ± 74.75	527.50 ± 95.89	t = 7.01 *P* < 0.0001*
**Six months postoperative** - Mean ± SD	915.25 ± 81.01	648.50 ± 117.32	t = 7.48 *P* < 0.0001*
**Repeated Measure ANOVA** ***P*** **-value**	*P* < 0.001*	*P* < 0.001*	
**Palatal Bone density**	**Group**		**Test of significance** ***P*** **-value**
	**Densah Group** **(n = 16)**	**Balloon Group** **(n = 16)**	
**Preoperative** - Mean ± SD	327.25 ± 72.02	342.00 ± 79.89	t = 0.548 *P* = 0.587 NS
**Immediate postoperative** - Mean ± SD	765.38 ± 72.16	631.25 ± 106.35	t = 4.17 *P* = 0.0002*
**Six months postoperative** - Mean ± SD	929.75 ± 75.90	760.38 ± 95.96	t = 5.53 *P* < 0.0001*
**Repeated Measure ANOVA** **P-value**	*P* < 0.001*	*P* < 0.001*	

SD.: Standard Deviation

t-test = independent t test

*Statistically significant (*P* ≤ 0.05)

NS: Statistically not significant (*P* > 0.05)

Regarding palatal bone density, there was no statistically significant difference in the palatal bone density around the implant between the two groups preoperatively (*P* = 0.587), but there was a statistically significant higher palatal bone density around the implant in Densah group compared with balloon group immediately postoperative and after 6 months (*P* = 0.0002, *P* < 0.0001), respectively (Table 7).

## Discussion

The rehabilitation of atrophic maxilla represents a challenge for oral and maxillofacial surgeon. Conventional implant treatments can’t be performed due to alveolar bone resorption and pneumatization of maxillary sinus. Since Bränemark discovered osseointegration [19], several surgical approaches for rehabilitating atrophic maxilla with dental implants have been proposed [20]. One of these approaches is maxillary sinus floor augmentation which is a predictable technique for obtaining the volumetric amount “vertical height” of bone required to place the implants [21, 22].

Sinus floor elevation can be executed via two distinct methods: the direct lateral window technique or the alternative indirect crestal method. The crestal approach to elevate the sinus floor is recognized for its minimally invasive nature, and its reduced complications when contrasted with the lateral window technique [23]. This method demonstrates enhanced predictability as a treatment option when the existing bone height is within the range of 5 to 6 mm, which is essential for ensuring primary stability [24].

The rationale of the present study was to compare the efficacy of Antral Membrane Balloon Elevation with Densah burs as minimally invasive techniques for atraumatic elevation of the sinus membrane through a transcrestal access.

The Antral Membrane Balloon Elevation (AMBE) technique is a minimally invasive approach, involves lifting the sinus membrane with minimal trauma, making it particularly advantageous for accessing challenging areas. Notably, it avoids sharp dissection around adjacent tooth roots, potentially reducing complications such as morbidity, blood loss, operative time, and postoperative pain compared to conventional methods [25]. The balloon technique can be applied to alveolar crests measuring 4 mm or less [8], in contrast to the classical indirect technique with osteotomes, where the minimum acceptable crest height is 6 mm [26].

A new surgical technique called osseodensification maintains bone by preparing the implant site by using Densah burs in anticlockwise direction (non-cutting motion) with copious irrigation. The osteotomy is gradually expanded in both the lateral and apical directions. The osseodensification concept is used for lateral expansion of narrow ridge, increase implant primary stability in low density bone and trasncresal sinus lifting procedure. The distinct flute design of Densah burs and counterclockwise rotation enable the lateral compaction of autogenous bone along the osteotomy walls and toward the sinus floor. This compaction, coupled with controlled pressure during drilling and a consistent in-and-out pumping motion, results in the creation of hydraulic pressure by well-hydrated autogenous bone, gently lifting and freeing the Schneiderian membrane [14, 15, 27, 28].

The choice of the bone grafting material to assist bone formation after sinus membrane elevation is still controversial [29]. None of the bone grafting materials on the market have all of the ideal properties as a bone substitute material [30, 31]. Autogenous bone grafting is widely recognized as the preferred method for grafting and reconstructive procedures in oral and maxillofacial surgery due to its established safety and reliability. However, it is also accompanied by several disadvantages, including the requirement for a second surgical site, heightened surgical risks, limited availability, and a relatively high rate of resorption [32].

In our study, allograft was used in both groups. Several studies have used allograft with maxillary sinus lifting procedures with simultaneous implant placement and had successful results [23, 24]. Allograft has osteoconductive properties, so it acts as a scaffold for bone formation [30, 33].

The survival rate in our study was 100% in both Groups. None of the cases showed any infection. Clinically and radiographically, all cases exhibited normal healing without any complications. Tang et al. [34] have reported that perforation of the Schneiderian membrane is the most common complication associated with transcrestal maxillary sinus lifting, occurring in 20% of cases. However, perforation was not reported in any case within the two groups, this could be attributed to the use of non-traumatic techniques, either by balloon or densah burs, which are capable of gentle slow elevation of sinus membrane.

Postoperative pain was examined using 10-point visual analogue scale (VAS) in the first 7 days. The two groups showed statistically significant decrease in pain score to reach score 0 by the end of the 7th day with no statistically significant difference between them. The pain experienced in both groups ranged from mild to moderate, consistent with findings reported by López-Quiles et al. [35].

Assessment of implant stability with the ISQ value of RFA is a non-invasive method widely used in the literature [36]. In this study, there was significant difference between the two groups in favor of Densah group at the time of implant placement (*P* = 0.004), but there was no significant difference between the two groups after 6 months (*P* = 0.07).

The notable disparity in primary stability between the two groups was largely attributed to the drilling protocol. In the Densah group, the spring back effect and elastic recoil of the bone on the implant surface following insertion resulted in an enhanced mechanical connection between the implant and the surrounding bone [37]. This finding aligns with the research of Huwais and Meyer [14], who noted that the osseodensification technique could lead to improved primary stability, a higher percentage of bone at the implant surface, and increased insertion and removal torques compared to the conventional drilling technique.

There was a significant difference when comparing implant stability immediately postoperative and at 6 months in the same group (*P* < 0.001). This is due to secondary stability establishment.

Our primary stability result was in line with Elghobashy et al. [37] and Hashem et al. [16], who compared the osseodensification technique with other techniques in crestal sinus lift procedures and found that the Densah group had the highest primary implant stability, but our result contradicts them regarding secondary stability.

Regarding residual bone height, it was almost the same in both groups, and there was no statistically significant difference between them (*P* = 0.624), indicating the homogeneity of the two groups at the start of the study.

In the present study, the mean immediate postoperative vertical bone height was 14.40 ± 0.53 mm in balloon group, while it was 12.70 ± 1.28 mm in Densah group, so there was a significant difference between the two groups in favor of balloon group (*P* < 0.0001) immediately postoperative, but there was no statistically significant difference between both groups after 6 months (*P* = 0.11).

Regarding the vertical bone gain in our study, the mean immediate postoperative vertical bone gain in balloon group and Densah group was 8.31 ± 0.44 mm and 6.75 ± 1.13 mm, respectively, so there was a significant difference between the two groups in favor of balloon group (*P* < 0.0001) immediately postoperative. After 6 months, there was no statistically significant difference between both groups (*P* = 0.25).

This is due to the fact that each 0.5 cc of the saline has an elevated sinus membrane of 6 mm in balloon technique [25], while Densah bur is used to pass the sinus floor and advance the height no more than 3 mm [38]. In addition, 0.5 cc of allograft was inserted in both groups which resulted in more elevation of the sinus membrane.

This was similar to López-Quiles et al. [35], and Abdalhameed et al. [25] who had 8.22 mm and 7.27 mm of immediate postoperative vertical bone gain, respectively after a crestal sinus lift procedure using antral membrane balloon elevation. Also, the result of our study was in line with Hashem et al. [16], who had 5.81 mm of vertical bone gain after crestal sinus floor elevation using Densah burs.

In contrast to our study, Ismaeil et al. [39], reported 4.55 mm of vertical bone gain after crestal sinus lift using balloon technique after 3 months of implant insertion.

The IPL appears to be a critical factor affecting new bone formation. Our study demonstrated that the mean IPL was 3.66 ± 0.88 mm in balloon group and 3.80 ± 1.07 mm in Densah group, with no statistically significant difference between the two groups (*P* = 0.689). When IPL is between 3 and 5 mm, new bone formation will be of a great amount. Our findings are consistent with those of Lin et al. [40].

Regarding reduction in vertical height, the mean was 2.28 ± 0.57 mm and 1.13 ± 0.40 mm in balloon group and Densah group, respectively, with statistically significant higher vertical height reduction in balloon group than Densah group (*P* < 0.0001). This could be attributed to the standardization of the quantity of bone graft for each implant at 0.5 cc allograft. In addition, the greater the elevation of the sinus membrane in balloon group, the greater the membrane tension will be, and the tension may be transferred to the force of compression on the grafting materials [41, 42].

Similar to our results, Jensen et al. [43], found that the resorption rate of bone is impacted by the types of graft materials. They observed resorption rates of 1.8 mm in autograft, 2.1 mm in demineralized allograft, 0.9 mm in alloplast, and 0.8 mm in autograft mixed with alloplast.

Regarding the bone density, there was no significant difference between the two groups in preoperative buccal and palatal bone density (*P* = 0.256, *P* = 0.587), respectively, which indicated the homogeneity of the two groups at the start of the study. And, throughout the study, the buccal and palatal bone density showed a significant increase from baseline to 6 months in the same group. This increase in bone density was related to the technique itself.

There was a significant difference in the buccal and palatal bone density between the two groups in favor of Densah group immediately postoperative and after 6 months. This is due to osseodensification technique using Densah burs cause motorized expansion of osteotomy site which results in increase the bone density around the implant. This is in line with other studies of Kumar et al. [44], and Huwais and Meyer [14]. This increase in bone density in Densah group is coincide with the high primary stability, where the stability depends on close contact between the implant surface and the surrounding bone [14].

The limitations of this study are a small sample size and a relatively short follow up period. An extended follow up period may yield a more pronounced result. So, greater sample sizes and longer follow-up periods should be the main goals of future research.

## Conclusion

Within the limitations of the current clinical trial, it can be concluded that Antral Membrane Balloon Technique and Osseodensification technique using Densah burs are successful minimally invasive methods for transcrestal sinus lift, providing excellent clinical and radiographic results without an increased risk of Schneiderian membrane perforation. Both techniques can be used for transcrestal sinus lift when RBH ranges from 4 to 7 mm. Antral Membrane Balloon technique demonstrated better immediate postoperative vertical bone height and vertical bone gain, and the Densah group demonstrated better implant primary stability, reduction in vertical bone height and bone density.

## Data Availability

The corresponding author can provide the data sets utilized and/or analyzed for this study upon reasonable request.

## Abbreviations

OSFE: Osteotome sinus floor elevation
AMBE: Antral membrane balloon elevation
IRB: The Institutional Review Board
SPSS: Statistical Package for the Social Sciences
RBH: Residual Bone Height
CBCT: cone-beam computed tomography
RFA: Resonance Frequency Analysis
ISQ: Implant stability quotient
VAS: Visual Analogue Scale
VBH: Vertical bone height
VBG: Vertical bone gain
IPL: Implant protrusion length
